# Supplementary material for: Novel statistically equivalent signature-based hybrid feature selection and ensemble deep learning LSTM and GRU for chronic kidney disease classification
Source: PeerJ Comput Sci. 2024 Nov 13;10:e2467. doi: 10.7717/peerj-cs.2467 (PMC11639220; doi:10.7717/peerj-cs.2467)
Supplement: Supplemental Information 3 [file peerj-cs-10-2467-s003.docx]

**1. Machine Learning Algorithms/Code**

- **Implementation Language:** Python
- **Libraries/Packages:** List the libraries and their versions (e.g., scikit-learn, TensorFlow, Keras, pandas).
- **Code Repository:**

**# Import necessary libraries**

**import numpy as np**

**import pandas as pd**

**from sklearn.model_selection import train_test_split**

**from sklearn.feature_selection import SelectKBest, f_classif**

**from sklearn.preprocessing import StandardScaler**

**from keras.models import Sequential**

**from keras.layers import Dense, LSTM, GRU**

**from keras.utils import np_utils**

**# Load your dataset**

**# Assuming you have a dataset stored in 'data.csv'**

**data = pd.read_csv('data.csv')**

**# Separate features and labels**

**X = data.drop('class', axis=1).values**

**y = data['class'].values**

**# Feature selection**

**selector = SelectKBest(score_func=f_classif, k=10) # Adjust k value as needed**

**X_selected = selector.fit_transform(X, y)**

**# Split data into training and testing sets**

**X_train, X_test, y_train, y_test = train_test_split(X_selected, y, test_size=0.2, random_state=42)**

**# Standardize features**

**scaler = StandardScaler()**

**X_train = scaler.fit_transform(X_train)**

**X_test = scaler.transform(X_test)**

**# Convert labels to categorical**

**y_train = np_utils.to_categorical(y_train)**

**y_test = np_utils.to_categorical(y_test)**

**# Define LSTM model**

**def create_lstm_model(input_shape):**

**model = Sequential()**

**model.add(LSTM(100, input_shape=input_shape))**

**model.add(Dense(2, activation='softmax'))**

**model.compile(loss='categorical_crossentropy', optimizer='adam', metrics=['accuracy'])**

**return model**

**# Define GRU model**

**def create_gru_model(input_shape):**

**model = Sequential()**

**model.add(GRU(100, input_shape=input_shape))**

**model.add(Dense(2, activation='softmax'))**

**model.compile(loss='categorical_crossentropy', optimizer='adam', metrics=['accuracy'])**

**return model**

**# Train LSTM model**

**lstm_model = create_lstm_model(X_train.shape[1:])**

**lstm_model.fit(X_train, y_train, validation_data=(X_test, y_test), epochs=10, batch_size=64)**

**# Train GRU model**

**gru_model = create_gru_model(X_train.shape[1:])**

**gru_model.fit(X_train, y_train, validation_data=(X_test, y_test), epochs=10, batch_size=64)**

**# Evaluate models**

**lstm_scores = lstm_model.evaluate(X_test, y_test, verbose=0)**

**print("LSTM Accuracy: %.2f%%" % (lstm_scores[1]*100))**

**gru_scores = gru_model.evaluate(X_test, y_test, verbose=0)**

**print("GRU Accuracy: %.2f%%" % (gru_scores[1]*100))**

**2. Computing Infrastructure**

- **Operating System:** Windows 10.
- **Hardware:** Intel i7, GPU (e.g., NVIDIA GTX 1080), and RAM (e.g., 16 GB).
- **Environment:** Conda environments

**3. README File**

# CKD Classification with Feature Selection and Deep Learning Models

This project involves preprocessing a dataset, selecting significant features, and using deep learning models (LSTM and GRU) to classify chronic kidney disease (CKD). The code includes data loading, preprocessing, feature selection, model training, and evaluation.

## Requirements

- Python 3.x

- numpy

- pandas

- scikit-learn

- keras

- tensorflow

## Installation

1. Clone the repository:

```bash

git clone https://github.com/username/ckd-classification.git

cd ckd-classification

**4. Reproduction Script**

#!/bin/bash

# Step 1: Set up the virtual environment

echo "Setting up the virtual environment..."

python3 -m venv ckd-env

# Step 2: Activate the virtual environment

echo "Activating the virtual environment..."

source ckd-env/bin/activate

# Step 3: Install required packages

echo "Installing required packages..."

pip install numpy pandas scikit-learn keras tensorflow

# Step 4: Ensure the dataset is available

if [ ! -f data.csv ]; then

echo "Error: data.csv not found in the project directory. Please place your dataset in the project directory and try again."

exit 1

fi

# Step 5: Run the classification script

echo "Running the CKD classification script..."

python ckd_classification.py

# Step 6: Deactivate the virtual environment

echo "Deactivating the virtual environment..."

deactivate

echo "Reproduction complete. Check the output above for model performance."

- **Single Script or Notebook:** Provide a script or Jupyter notebook that can reproduce the key results of the study from start to finish.
- **Automated Execution:** Ensure the script is automated as much as possible, requiring minimal manual intervention.
- **Random Seeds:** Set and document any random seeds used to ensure reproducibility of results.

**5. Data Preprocessing**

**## Data Preprocessing**

**### Handling Missing Values**

**The dataset contains missing values that need to be addressed before model training. The following steps were taken to handle these missing values:**

**1. **Identification:** All missing values (NaNs) in the dataset were identified.**

**2. **Removal:** Rows containing missing values were removed from the dataset to ensure that the data is clean and complete for model training.**

**This approach was chosen to maintain simplicity and avoid potential biases introduced by imputation methods.**

**### Example Code for Handling Missing Values**

**The following Python code snippet demonstrates how missing values were removed from the dataset:**

**```python**

**import pandas as pd**

**# Load the raw data**

**data = pd.read_csv('data/raw_data.csv')**

**# Identify and remove rows with missing values**

**data_cleaned = data.dropna()**

**# Save the cleaned data (optional)**

**data_cleaned.to_csv('data/cleaned_data.csv', index=False)**

**6. Description of Models Used**

This study proposes the Least Absolute Shrinkage and Selection Operator (LASSO) and SES feature selection approach for CKD feature identification. Later, A combination of the Long short-term memory (LSTM) and Gated Recurrent Unit (GRU) ensemble deep-learning model is proposed for the CKD classification task. The features selected by the hybrid feature selection method are input into an ensemble deep-learning model

**7. Assessment Metrics**

Evaluation metrics such as accuracy, precision, recall, and F score assess the model’s performance. The experimental results are compared with individual classifiers such as Decision tree (DT), Random Forest(RF), Logistic Regression (LR), and Support Vector Machine(SVM ). The results show a 2% improvement in classification accuracy when considering the proposed hybrid feature selection approach and Ensemble Deep Learning model LSTM and GRU.

**8. Limitations/Validity**

The results show a 2% improvement in classification accuracy when considering the proposed hybrid feature selection approach and Ensemble Deep Learning model LSTM and GRU. Further analysis indicates that certain features, including HEMO, POT, bacteria, and coronary artery disease, contribute minimally to classification tasks.
